# Termination, Advancement, and Delaying Responses to His Synchronous Premature Ventricular Contractions During Narrow QRS Tachycardia: What Are the Possible Mechanisms?

**DOI:** 10.19102/icrm.2021.120108

**Published:** 2021-01-15

**Authors:** Ahmet Korkmaz, Meryem Kara, Ozcan Ozeke, Serkan Cay, Firat Ozcan, Serkan Topaloglu, Dursun Aras

**Affiliations:** ^1^Department of Cardiology, Ankara City Hospital, University of Health Sciences, Ankara, Turkey

**Keywords:** Atrioventricular nodal reentrant tachycardia, bystander, His-refractory ventricular premature complex, slow pathway, supraventricular tachycardia

## Abstract

The differential diagnosis of a regular, narrow QRS, long-R–P tachycardia includes atypical atrioventricular nodal reentry tachycardia, atrial tachycardia, and atrioventricular reentry tachycardia via a slowly conducting accessory pathway with decremental conduction properties. Almost all described diagnostic maneuvers in the electrophysiology laboratory have exceptions to their primary interpretation. The usual proviso is that the observation must be reproducible.

## Case presentation

**Figure 1:**
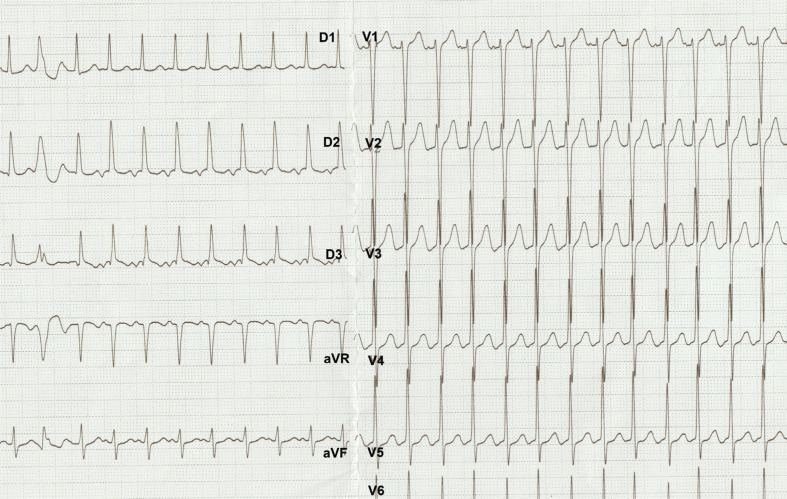
A narrow QRS tachycardia easily induced by a PVC.

**Figure 2:**
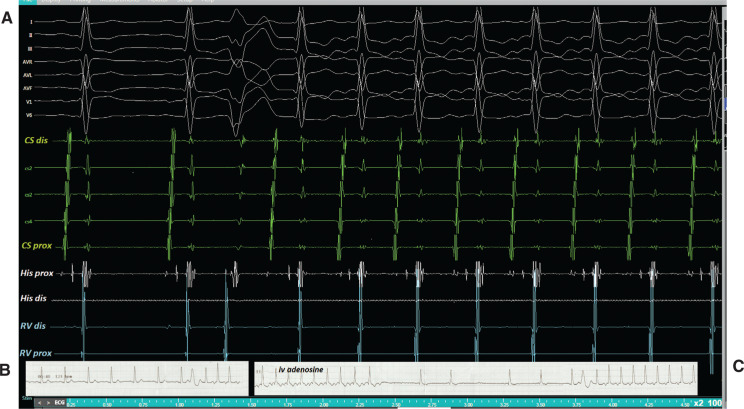
The tachycardia was easily induced by PVCs during EP study **(A)** and electrocardiography monitoring **(B)**. **C:** The intravenous adenosine terminated the tachycardia with a retrograde P-wave, which then easily started up again with a PVC, showing an incessant nature.

**Figure 3:**
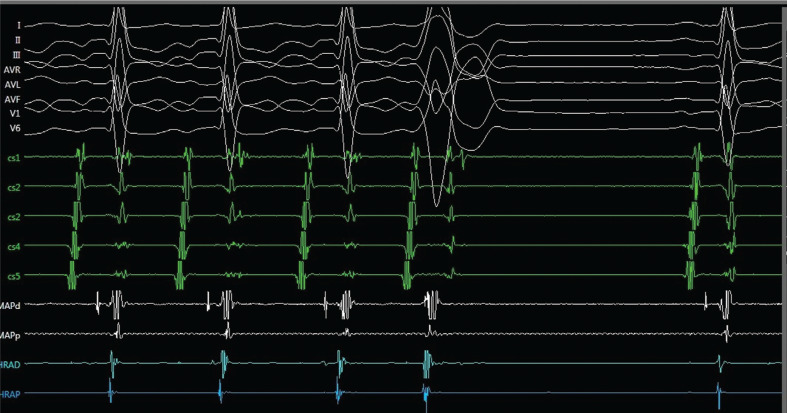
A spontaneous His-refractory PVC during the tachycardia terminated the tachycardia without retrograde atrial activation.

A 27-year-old female with a history of narrow complex tachycardia was referred for radiofrequency ablation. Her tachycardia was easily induced by premature ventricular contractions (PVCs) during electrocardiography **(Figure 1)** and electrophysiology (EP) study **(Figure 2)**. A single spontaneous His-refractory PVC (Hr-PVC) during the tachycardia terminated the ongoing tachycardia **(Figure 3)**. What is the possible mechanism of this termination response of tachycardia?

## Discussion

The differential diagnosis of a long-R–P supraventricular tachycardia with the earliest atrial activation in the His-bundle region includes atypical atrioventricular (AV) nodal reentry tachycardia (AVNRT), atrial tachycardia (AT), AV reentry tachycardia (AVRT) via a slowly conducting accessory pathway with decremental conduction properties, nodofasciular reentrant tachycardia (NFRT), or nodoventricular reentrant tachycardias (NVRT).^1–6^

Post-PVC prolongation of the P–R interval and tachycardia initiating can be explained by concealed conduction. The partial penetration of an impulse into the AV node after a PVC might infer the behavior of the subsequent sinus impulse that is conducted through the AV node **(Figures 2A and 2B)** for ease of initiation of AV node– dependent tachycardias.^7,8^ Adenosine-induced termination of this tachycardia, with an A-wave followed by A–H block, eliminated the diagnosis of AT **(Figure 2C)**.^4^ An Hr-PVC in **Figure 3** stopped the tachycardia without retrograde conduction to the atrium, excluding the diagnosis of AT^3,9,10^ and leaving us with the possibility of AVNRT with a bystander nodofasciular pathway.^4,11–14^ Termination of the tachycardia with an Hr-PVC without affecting atrial depolarization is suggestive of AVRT or NFRT/NVRT^3,15^; however, the coincidental termination or atypical AVNRT with bystander pathways might also explain this response.^16^ In the current case, the termination response without A (the atrial electrogram) was not reproducible. Almost all described diagnostic maneuvers in the EP laboratory have exceptions to their primary interpretation^17^; therefore, the usual proviso is that the observation must be reproducible.

**Figure 4:**
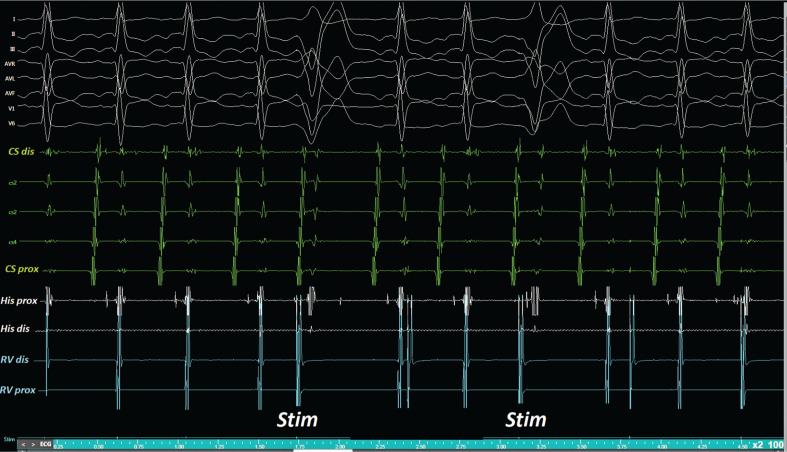
Whereas the atrial activation seems “delayed” without a change in the atrial activation sequence after the first Hr-PVC, it appears “advanced” without a change in the atrial activation sequence after the second Hr-PVC.

**Figure 5:**
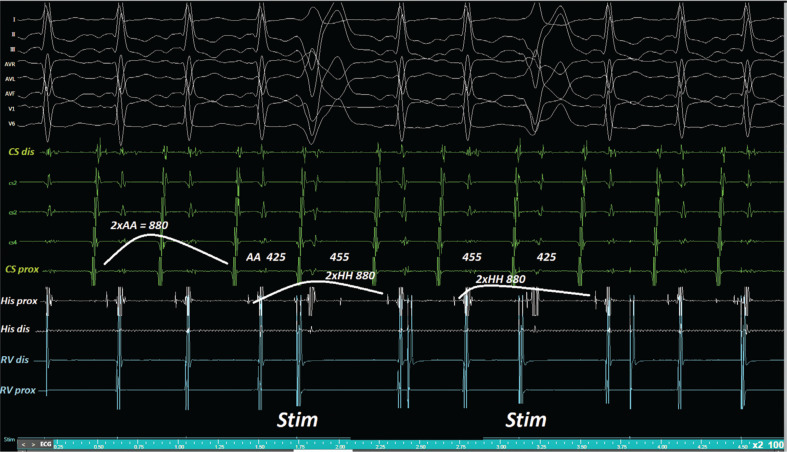
Two H–H intervals were each equal to double the length of an H–H interval for both Hr-PVC cycles (based on measurement of the interval lengths of the two H–H intervals immediately before and after the Hr-PVCs).

**Figure 6:**
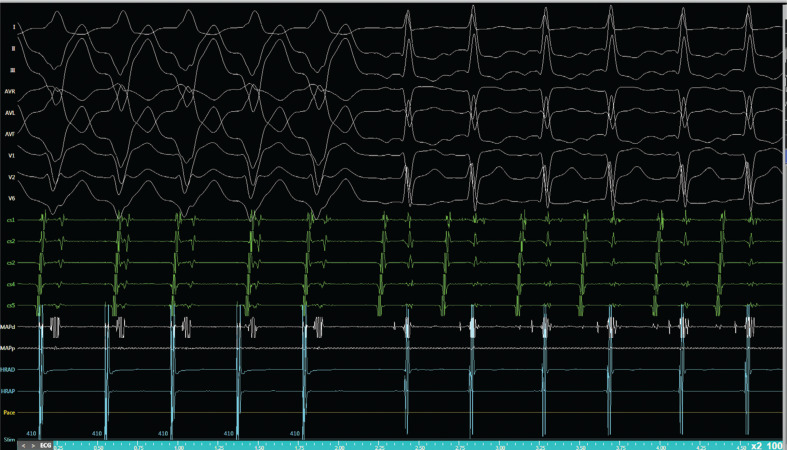
Ventricular overdrive suppression shows no entrainment but suggests dissociation of the ventricle from the ongoing tachycardia. The V-A-H-V response also excludes an AT.

It was interesting that both advancement-like and delay-like responses to Hr-PVCs were observed during the current case **(Figure 4)**. However, the cycle length (CL) alternation during tachycardia was of such a large degree that it should be readily identifiable. Therefore, the Hr-PVC response in the current case was not applicable as an EP maneuver. As the durations of two H–H intervals were each equal to double the length of an H–H interval for both Hr-PVC cycles (based on measurement of the interval length of the two H–H intervals immediately before and after the Hr-PVC), both Hr-PVC responses were accepted as pseudo-resetting responses (ie, an “advancement-like response” to the first Hr-PVC and “delayed-like response” to the second Hr-PVC) **(Figures 4 and 5)**, which can be explained by a reciprocal CL alternans during AVNRT. Ho et al. reported that Hr-PVC assessment is the only viable maneuver by which to identify a concealed, bystander NFRT during atypical AVNRT^18^; these pseudo-resetting responses to Hr-PVCs also favor atypical AVNRT over NFRT/NVRT.^18^ Furthermore, the ventricular overdrive suppression did not entrain the tachycardia but rather dissociated the ventricle from the ongoing tachycardia, ruling out the AVRT or nodoventricular pathway as a tachycardia mechanism **(Figure 6)**.^14,19,20^ These results collectively seem sufficient to establish a diagnosis of atypical AVNRT. A longitudinal dissociation of one slow pathway or two separate slow pathways could explain the oscillation of two CLs.^21^ The degree of wobble in this example is about 40 ms, raising the possibility of atypical AVNRT using two separate slow pathways for retrograde conduction.^22^ Slow pathway ablation was successful in eliminating the arrhythmia. This case stresses that almost all described diagnostic maneuvers in the EP laboratory have exceptions to their primary interpretation.^17^
